# Development and validation of a risk perception scale on acute respiratory infections for caregivers in long-term care facilities

**DOI:** 10.3389/fpubh.2025.1527905

**Published:** 2025-05-02

**Authors:** Zhihao Xie, Jiayi Zhu, Xiaofeng Xie, Lei Huang, Jianhua Ren, Ruben Martin-Payo, Mengying Qiu, Fengying Zhang

**Affiliations:** ^1^Nursing Key Laboratory of Sichuan Province, West China School of Nursing, West China Hospital, Sichuan University, Chengdu, Sichuan, China; ^2^Department of Dermatology and Venerology, West China Hospital, Sichuan University, Chengdu, Sichuan, China; ^3^Department of Nursing, West China Second University Hospital, Sichuan University, Chengdu, Sichuan, China; ^4^Key Laboratory of Birth Defects and Related Diseases of Women and Children (Sichuan University), Ministry of Education, Chengdu, Sichuan, China; ^5^Faculty of Medicine and Health Sciences, Universidad de Oviedo, Oviedo, Spain; ^6^PRECAM Research Group, Health Research Institute of Asturias (ISPA), Oviedo, Spain; ^7^School of English Studies, Sichuan International Studies University, Chongqing, China

**Keywords:** risk perception, acute respiratory infection, caregiver, long-term care facility, scale development

## Abstract

**Introduction:**

Acute respiratory infections (ARIs) represent a significant threat to global public health, particularly among older adults residing in long-term care facilities (LTCFs), where high-density living conditions facilitate the rapid transmission of infections. The perception of risk regarding ARIs among caregivers is critical, as it directly influences their protective behaviors and decision-making during epidemic outbreaks. Despite the importance of this perception, there is currently no validated instrument specifically designed to assess caregivers’ risk perception of ARIs within the context of LTCFs. This study aims to address this gap by developing a reliable and accessible scale to measure caregivers’ risk perception.

**Methods:**

We developed an initial scale through a comprehensive literature review and two rounds of Delphi consultations with 19 experts in related field. A total of 428 participants in LTCFs were surveyed, yielding 343 valid responses and efficiency rate of 80.14%. Following a systematic scale development process that encompassed pretest, item analysis, and exploratory factor analysis (EFA) involving 123 respondents, we refined the scale to its robustness. Confirmatory factor analysis (CFA) was conducted with an additional 220 participants, alongside rigorous tests for reliability, stability, and validity, to evaluate the final scale.

**Results:**

The developed scale consists of nine items categorized into three dimensions: severity, controllability, and susceptibility, all of which meet essential criteria for reliability and validity. The overall Cronbach’*α* coefficients for the scale was 0.795, with each dimension coefficient of 0.795, 0.707, and 0.791, respectively.

**Discussion:**

In its current form, this scale serves as a valuable tool for managers and practitioners, enabling them to preliminarily assess caregivers’ risk perceptions regarding ARIs in LTCFs. By enhancing our understanding of caregivers’ behaviors, this instrument has the potential to inform targeted interventions.

## 1 Introduction

Acute respiratory infections (ARIs) represent a major worldwide health problem associated with high morbidity and mortality Diseases GBD (1), which poses a serious threat to human beings globally. As a public health problem, ARIs have contributed to numerous deaths and huge financial losses (2, 3). The most notable recent public health crisis, the COVID-19 pandemic, has led to over 153 million cases of disability (4) and approximately 5 million deaths (5). This underscores the urgent need for effective prevention and management strategies to mitigate the impact of ARIs on populations globally.

Controlling sources of infections, interrupting routes of transmission, and protecting susceptible populations are the three most crucial strategies for the preventing ARIs. Among all susceptible populations, individuals aged 65 years and older are particularly vulnerable (5). Compared to other age groups, older adults not only exhibit a higher susceptibility to ARIs but are also more likely to experience severe complications during the ARIs epidemics (6–9).

In long-term care facilities (LTCFs), where older adults are concentrated, the risk of ARI transmission is significantly heightened. Many residents in LTCFs also suffer from conditions such as dementia, strokes, or other chronic diseases, which may mask the symptoms of ARIs and delay the implementation of effective preventive measures (10). Therefore, LTCFs require broader concerns in the prevention and control of ARIs to safeguard the health of this vulnerable population.

Risk perception was first introduced by Raymond Bauer in 1960, who defined it as the consumer’s perception of uncertainty regarding the outcomes of purchasing decisions (11). In the realm of public health, risk perception is closely linked to the adoption of healthy behaviors (12–14). It plays a crucial role in the individual decision to take preventive actions (15).

For caregivers, their perception of risk significantly influences their care behaviors, which directly affect disaster risk prevention for older adults inhabiting LTCFs. Risk perception encompasses the public’s intuitive judgments about risks (16), and it could shape individuals’ coping behavior and then contributes to the adoption of protective measures while facing risks (17).

Additionally, individual risk perception not only aids in the formulation of public health interventions, but also impacts the implementation and effectiveness of those interventions (18, 19). Therefore, assessing caregivers’ risk perception regarding ARIs is crucial for managers and researchers for LTCF. This understanding enables them to evaluate potential risks and design effective training programs or interventions for caregivers. Ultimately, enhancing caregivers’ awareness and understanding of ARI risks contributes to the prevention of ARIs outbreaks and helps maintain the well-being of older adults in these facilities.

However, existing scales measuring risk perception related to ARIs have not specifically addressed the perception of caregivers in LTCFs. Recognizing the uniqueness and importance of caregivers’ risk perception, this study aims to develop an available instrument for caregivers to assess their risk perception of ARIs among the older adults they care for in LTCFs. This instrument will be essential for effective risk management within these facilities, providing valuable insights that can guide training and intervention strategies to enhance the safety and well-being of residents.

## 2 Methods

### 2.1 Research procedures

**Figure 1:**
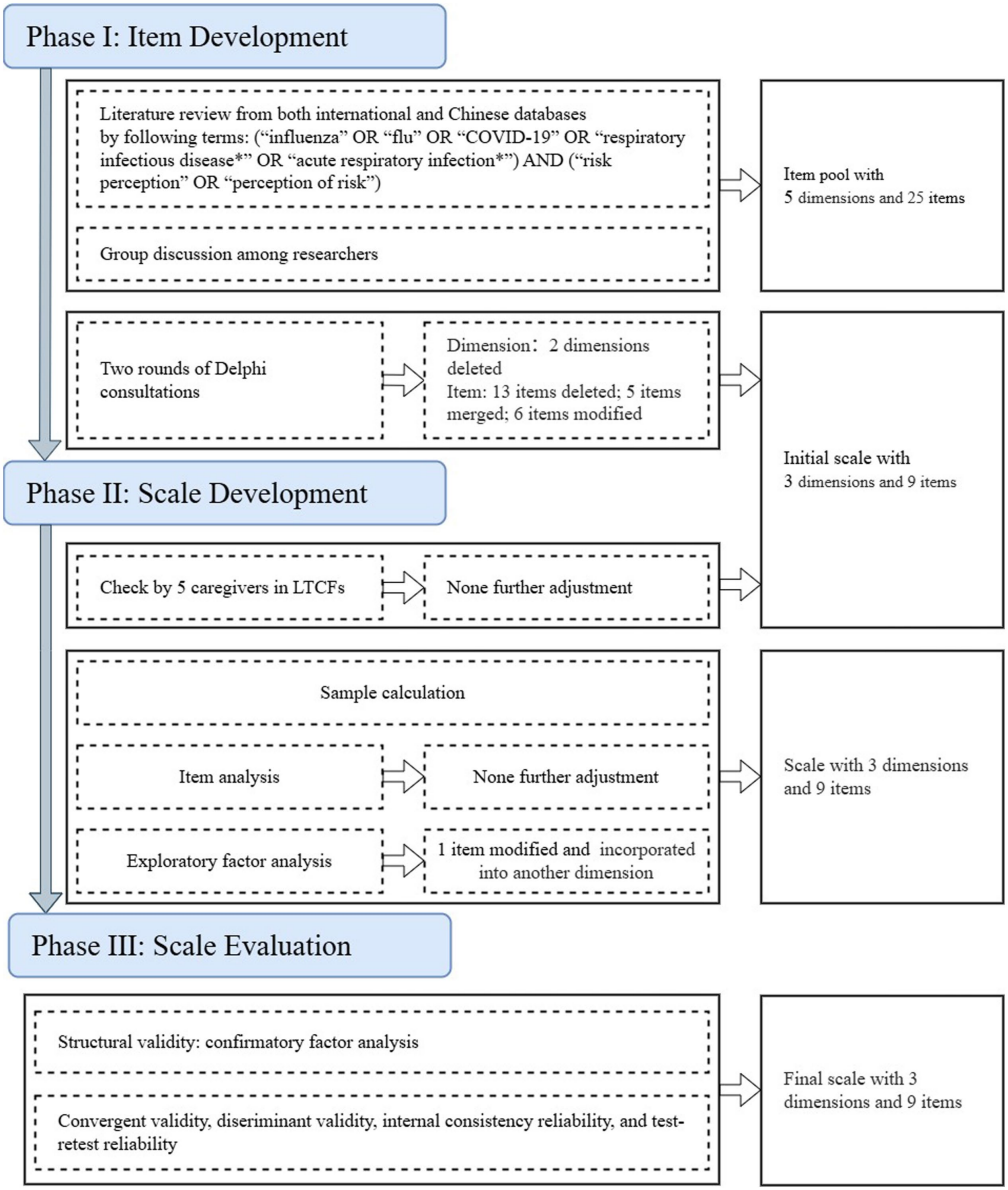
Study flow.

This study adhered to the recommended “three phases and nine steps of scale development and validation” procedure (20) in the development of risk perception scale on ARI for caregivers in LTCFs (RPSACL) (Figure 1).

#### 2.1.1 Construction of item pool

The initial scale was formed based on extensive literature review and Delphi consultation method. Firstly, we searched literature from database establishment to September 30, 2021 in English and Chinese using the following terms: (“influenza” OR “flu” OR “COVID-19” OR “respiratory infectious disease*” OR “acute respiratory infection*”) AND (“risk perception” OR “perception of risk”). We identified 863 correlated studies and only 7 of those studies developed scales that are relevant (21–27). Initial Item pool was developed (Table 1) based on a comprehensive literature review and discussion between research team members.

**Table 1:** Item pool.

Dimension	Items
1. Individual risk perception	1.1 I attach importance to the prevention of acute respiratory infections.
	1.2 I think I have the impossibility to catch acute respiratory infections.
	1.3 I am worried about infected with acute respiratory infections.
	1.4 I feel a great risk from acute respiratory infections.
2. Severity	2.1 Older adults will suffer a lot if infected by acute respiratory infections.
	2.2 Older adults will have serious sequelae if infected.
	2.3 Older adults will face a greater risk of death if infected by acute respiratory infections.
	2.4 The detection and treatment of acute respiratory infections will be delayed if virus mutation happened.
	2.5 The signs of infection in older adults are difficult to detect.
	2.6 It will cause serious impact if the acute respiratory infections epidemic occurred in LTCF.
3. Controllability	3.1 It will spread rapidly if acute respiratory infections occurred in LTCF.
	3.2 It will be difficult to cure if older adults were infected by acute respiratory infections.
	3.3 Older adults in LTCFs are still facing the risk of acute respiratory infections.
	3.4 The risk management will be difficult, if acute respiratory infections occurred in LTCF.
	3.5 Older adults are impossible to be infected because LTCFs have been ready to prevent and control acute respiratory infections.
4. Susceptibility	4.1 Older adults are the most likely to be infected.
	4.2 Older adults are the most likely to be infected than any other populations.
	4.3 The preventive measurements taken by our facility are sufficient to prevent older adults from infected by acute respiratory infections.
	4.4 The negligence may happen even if the preventive measures are strictly taken.
5. Coping behavior	5.1 I will wear a mask when approaching people outside or go out of the facility.
	5.2 I will wash my hands before and after touching older adults.
	5.3 I will reduce the use of public transport.
	5.4 I will stay away from crowded places.
	5.5 I have ever postponed or canceled unnecessary social events.
	5.6 I reduced the opportunities to go out.

#### 2.1.2 Delphi method

After forming the item pool, the Delphi method was conducted involving 10–20 experts in LTCF management, geriatric nursing, and risk management (28, 29). Experts were required to assess the importance and feasibility of every dimension and item by scoring. This approach was designed to ensure a broad range of perspectives while maintaining manageability. Invitation letters containing the initial scale were sent to the experts via email, which included clear instructions for participation. Each expert completed the questionnaire and returned their feedback within a 14-day timeframe. This iterative process continued until a consensus was reached or until there were no significant changes in the responses. The primary task of experts was to evaluate the importance and feasibility of each dimension and item within the scale. They utilized a scoring system ranging from 0 to 5 points for their assessments. In addition to providing these ratings, experts were encouraged to suggest modifications to enhance each item. To ensure the reliability of the consultation, expert authority coefficients were calculated based on academic standard weight, judgment weight, and familiarity weight (Supplementary material 1). According to established guidelines, an expert authority coefficient greater than 0.7 indicates a reliable consultation process (30). The items whose feasibility or importance scores have a maximum score ratio of over 80% and a coefficient of variation over 25% was the preliminary screening principle for each dimension and item (29). At the conclusion of the Delphi consultation questionnaire, the experts reflected on their judgments and assessed their familiarity with the content being evaluated. This reflective component allowed for a deeper understanding of their perspectives and ensured that their insights were well-informed. Throughout the consultation, we engaged in a critical evaluation of the scale’s dimensions and items, leveraging our collective expertise to refine and validate the tool effectively.

#### 2.1.3 Pre-test

To make sure each item could be well understanded by respondents, pre-test was conducted on caregivers in LTCFs after Delphi method. Necessary adjustment would be made if any item were not concise and understandable according to responders’ feedback. Then the scale was used to verify its reliability and validity.

#### 2.1.4 Sample calculation

We calculated the sample size with more than 10 times the number of items, which was recommended by Nunnally (31). So, we collected two groups of samples, one for item analysis and exploratory factor analysis, and the other for confirmatory factor analysis, as well as assessing Cronbach’*α* coefficient, test–retest reliability, and construct validity.

#### 2.1.5 Item analysis

We employed item discrimination and a homogeneity test to conduct item analysis. Conceptually, items within a scale should effectively differentiate between subjects in favorable and unfavorable conditions. Moreover, items designed to measure the same construct should share similar characteristics. We ranked participants based on their scores on the scale and classified the top 27% as the high group, while the bottom 27% as the low group (32). We then carried out independent samples *t*-tests to identify significant differences between these two groups (20). Items that did not exhibit statistically significant differences were deemed to lack discriminatory power and were removed from further analysis. For the homogeneity test, we calculated the correlation coefficients between items. Items with the absolute *t*-values were more than three indicated insufficient homogeneity (20). After completing the item analysis, only those items demonstrating both discriminative ability and homogeneity were retained for subsequent factor analysis.

#### 2.1.6 Exploratory factor analysis

Kaiser-Meyer-Olkin (KMO) test was conducted to determine whether the sample is qualified for exploratory factor analysis (EFA). The sample whose cut-off value was larger than 0.50 and the Bartlett’s test reaches a significant level was qualified for EFA (33). The maximum variance method was used in EFA. Items with factor loadings larger than 0.55 were included (32). Besides, the scale was proved to have good univariance contribution rate of more than 60% (34).

#### 2.1.7 Scale evaluation

Based on the result of EFA, we conducted confirmatory factor analysis (CFA) via structural equation modeling (SEM). Due to the sample size calculated in this research was less than 500, and questionnaires were scored as continuous variables, we conducted the generalized least square (GLS) method in this study (32). We adopted the following indicators to evaluate the model fit. Several indices of the CFA were retained to examine the model:

(1) χ^2^/degrees of freedom (χ^2^/DF): When the χ^2^/DF value is close to 1, it suggests that the model exhibits a strong explanatory power for the data. When this value falls between 1 and 3, the model fit can be considered moderate. However, when it is greater than 3, it is generally regarded as an indication that the model does not fit the data well, and it may be necessary to review and potentially modify the model (35).
(2) Normed Fit Index (NFI), Incremental Fit Index (IFI), and Comparative Fit Index (CFI): the values of these indexes are greater than 0.90 is considered acceptable (36).
(3) Root Mean Square Error of Approximation (RMSEA): When the model exhibits good fitness, the RMSEA is less than 0.05. Conversely, when the model fitness is poor, the RMSEA is higher than 0.1, indicating that modification is required. If the RMSEA value lies between 0.050 and 0.100, it implies that while the model is not highly satisfactory, it is still acceptable (36).

For reliability, we calculated the Cronbach’s *α* coefficient of the RPSACL and each dimension. The reliability was considered to be high when the Cronbach’s α coefficient was over 0.70 (37). To evaluate retest reliability of RPSACL, we selected 59 participants to evaluate their retest reliability after an interval of 4 weeks (38).

In order to verify the construct validity, we calculated the convergent validity and discriminant validity. Convergent validity mainly focuses on the degree of consistency among different measurement indicators within the same construct of a scale. Generally, when the composite reliability (CR) value is greater than 0.7, it indicates that the scale has good internal consistency (39). Discriminant validity aims to examine whether the scale can distinguish between different constructs. When the square root of the Average Variance Extracted (AVE) for a particular construct exceeds the correlation coefficients between said construct and others, and concurrently, its Composite Reliability (CR) value is greater than 0.7, it provides substantial evidence that the construct can be distinctly discriminated from other constructs. This indicates effective discriminant validity, highlighting the scale’s ability to distinguish between different constructs (39).

### 2.2 Sampling

Participants who meet the following criteria were included: (1) provide care for older adults; (2) work in LTCF; (3) have worked over 6 months; (4) fully understand and voluntarily participate in this study. Besides, those who meet the following criteria were excluded: (1) have resigned or retired; (2) refuse to participate in this survey.

A wide-ranging online survey was conducted among caregivers in LTCFs in China. Participants recruited were invited to fill out the online questionnaire connected to the Wenjuanxing platform^1^ after informed consent was given. It was guaranteed that their answer and private information would be confidential and processed anonymously.

### 2.3 Study instruments

This study utilized the questionnaires consisting of two parts. One was general demographic data questionnaire, including gender, age, education, marital status, working years in LTCF, charge of the LTCF, and another was the initial RPSACL. The initial RPSACL contains nine items, which measured risk perception from severity, controllability, and susceptibility. Besides, a five-point Likert scale was taken to score individuals’ level of risk perception. Items were assessed on a five-point Likert scale (1 = strongly disagree; 2 = disagree; 3 = neither agree nor disagree; 4 = agree; 5 = strongly agree), with higher score indicating higher level of risk perception. The total score ranged from 5 to 45 points.

### 2.4 Statistical methods

The data was analyzed by IBM SPSS 26.0 and IBM AMOS 25.0. Items analysis and exploratory factor analysis were taken to filter items. Besides, the reliability of the RPSACL was tested by calculating Cronbach’s *α* coefficient and stability was tested by Pearson correlation analysis. Hence, structural equation modeling was employed to regress RPSACL dimensions (using the validated RPSACL) on the identified risk perception factors.

### 2.5 Common method bias

Common method bias may influence the validity and reliability of the research results (40). To address this issue, we implemented several strategies aimed at mitigating its effects (41). First, we ensured anonymity and confidentiality for respondents, which is crucial for reducing social desirability bias and fostering more honest and reflective responses. Additionally, we incorporated attention-check questions in the survey to identify participants who may not be responding thoughtfully, allowing us to exclude inattentive responses from our analysis. Furthermore, we utilized statistical techniques, such as confirmatory factor analysis, to detect and control for the presence of common method bias within our dataset (42). These strategies aimed to enhance the overall quality of the data collected and ensure more reliable research outcomes.

### 2.6 Ethical considerations

This study has been approved by Ethics Committee on Biomedical Research, West China Hospital of Sichuan University [2020 Review (No. 1270)]. To ensure the informed consent of respondents and their voluntary participation, each respondent was required to review and sign the informed consent form prior to providing responses.

## 3 Results

### 3.1 Delphi consultation

We invited 19 experts majoring in LTCF management, geriatric nursing, and risk management as an expert panel (Table 2). Two rounds of Delphi consultations were conducted in total, during which consensus was ultimately achieved. A total of 19 consultation questionnaires were distributed in each round, and the effective recovery rate of expert consultation questionnaires consistently remained at 94.74%, indicating the comprehensive collection and analysis of data (43).

**Table 2:** Information of experts panel.

Field of experts	First round		Second round	
	Count	%	Count	%
LTCF management	6	37.84%	6	35.29%
Geriatric nursing	7	37.84%	7	41.18%
Risk management	5	24.32%	4	23.53%

In the first round of Delphi consultation, the overall expert authority coefficient for this study was calculated to be 0.85, with individual authority coefficient for valid experts ranged from 0.75 to 0.93 (Table 3). Detailed information regarding the dimensions of the questionnaire and the scores of each item can be found in Table 4. Then we engaged in-depth discussions with every expert to decide the revisions of the items according to the suggestions given by experts. Based on their input and the scores in expert questionnaire, we made revisions to the items and dimensions (Table 5).

**Table 3:** Experts authority coefficient.

No.	q1	q2	q3	Total	Authority coefficient
1	1.00	0.80	0.80	2.60	0.87
2	1.00	0.95	0.80	2.75	0.92
3	0.40	0.85	1.00	2.25	0.75
4	1.00	0.80	1.00	2.80	0.93
**5**	**0.40**	**0.80**	**0.80**	**2.00**	**0.67**
6	0.60	0.85	1.00	2.45	0.82
7	0.60	0.80	1.00	2.40	0.80
8	1.00	0.85	0.80	2.65	0.88
9	0.60	0.85	0.80	2.25	0.75
10	0.80	0.90	0.80	2.50	0.83
11	0.80	0.85	0.80	2.45	0.82
12	1.00	0.80	0.80	2.60	0.87
13	0.40	0.90	1.00	1.90	0.77
14	0.60	0.85	1.00	2.45	0.82
15	1.00	0.80	0.80	2.60	0.87
16	0.60	0.95	1.00	2.55	0.85
17	0.80	0.90	1.00	2.70	0.90
18	0.80	0.90	1.00	2.70	0.90
19	0.80	0.85	1.00	2.65	0.88
Total^①^	13.80	15.45	16.40	45.25	0.85

① Excluding expert no.5.

**Table 4:** Result of the first round consultation.

Dimensions	Item	Mean		SD		CV (%)		Full score ratio (%)	
		Feasibility	Importance	Feasibility	Importance	Feasibility	Importance	Feasibility	Importance
1. Individual risk perception		4.59	5.00	0.62	0.00	0.13	0.00	68.75	100.00
	1.1	4.88	4.94	0.33	0.25	6.81	5.06	83.33	83.33
	1.2	3.53	4.00	1.72	1.51	48.79	37.80	38.89	50.00
	1.3	4.71	4.63	0.60	0.72	12.79	15.54	72.22	66.67
	1.4	4.25	4.31	1.36	1.29	32.09	29.83	66.67	55.56
2. Severity		4.59	4.65	0.62	0.61	0.13	0.13	68.75	75.00
	2.1	4.81	4.81	0.39	0.38	8.17	7.97	83.33	83.33
	2.2	4.33	4.28	0.93	1.13	21.50	26.36	61.11	61.11
	2.3	4.39	4.63	1.01	1.14	22.95	24.60	66.67	66.67
	2.4	4.22	4.13	1.20	1.11	28.43	27.02	61.11	55.56
	2.5	3.88	3.88	1.15	1.19	29.56	30.58	27.78	33.33
	2.6	4.71	4.73	0.68	0.56	14.52	11.88	77.78	77.78
3. Controllability		4.41	4.71	0.80	0.59	0.18	0.12	62.50	81.25
	3.1	4.78	4.88	0.44	0.38	9.15	7.87	77.78	83.33
	3.2	4.39	4.56	0.87	1.18	19.83	25.83	55.56	61.11
	3.3	4.28	4.56	0.86	0.86	20.15	18.75	50.00	61.11
	3.4	4.22	4.50	1.25	0.79	29.64	17.46	61.11	66.67
	3.5	3.89	4.44	1.32	1.07	33.87	24.20	38.89	55.56
Susceptibility		4.47	4.65	0.87	0.70	0.20	0.15	68.75	75.00
	4.1	4.67	4.69	0.70	0.57	15.04	12.26	77.78	77.78
	4.2	4.56	4.60	0.91	0.82	19.95	17.75	66.67	61.11
	4.3	4.56	4.88	0.62	0.32	13.57	6.63	61.11	88.89
	4.4	4.47	4.40	0.89	0.89	19.95	20.27	66.67	55.56
Coping behavior		4.82	4.88	0.39	0.33	0.08	0.07	87.50	87.50
	5.1	4.89	4.94	0.24	0.24	4.96	4.77	88.89	94.44
	5.2	4.82	4.88	0.34	0.33	7.08	6.81	77.78	83.33
	5.3	4.67	4.75	0.59	0.55	12.60	11.54	72.22	83.33
	5.4	4.29	4.56	1.14	1.00	26.51	22.00	55.56	72.22
	5.5	4.71	4.53	0.58	0.87	12.27	19.20	72.22	72.22
	5.6	4.94	4.80	0.00	0.53	0.00	11.01	88.89	83.33

**Table 5:** Adjustment of items and dimensions.

Dimension	Item	Modification	Reason	Result
1. Individual risk perception		Delete	1. Overlapping with the connotations of other dimensions; 2. It does not belong to the risk of acute respiratory infections infection in older adults	
	1.1 I attach importance to the prevention and control of acute respiratory infections.	Modify	This entry conflicts with the role of the responders during the ARI epidemic. because the role of responders in epidemic prevention and control should be to take measures to prevent acute respiratory infections, while not to control it	1.1 I attach importance to the prevention of acute respiratory infections
	1.2 I think I have the impossibility to catch acute respiratory infections	Delete	Not meeting the initial screening conditions	
	1.3 I am worried about infected with acute respiratory infections	Delete	Not meeting the risk perception definition for caregivers in this study	
	1.4 I feel a great risk from acute respiratory infections	Delete	Not meeting the initial screening conditions	
2. Severity		Retain		1. Severity
	2.1 Older adults will suffer a lot if infected by acute respiratory infections	Merge	Repetitive content	1.2 Older adults will face the risk of death if infected by acute respiratory infections
	2.2 Older adults will have serious sequelae if infected	Merge	Repetitive content	
	2.3 Older adults will face a greater risk of death if infected by acute respiratory infections	Merge	Repetitive content	
	2.4 The detection and treatment of acute respiratory infections will be delayed if virus mutation happened	Delete	Not meeting the initial screening conditions	
	2.5 The signs of infection in older adults are difficult to detect	Modify	Likely to generate ambiguity	1.4 The symptoms of infection in older adults are more severe than in other age groups
	2.6 It will cause serious impact if the acute respiratory infections epidemic occurred in LTCF	Modify	Likely to generate ambiguity	1.3 acute respiratory infections epidemic will contribute grave consequences
3. Controllability		Retain		2. Controllability
	3.1 It will spread rapidly if acute respiratory infections occurred in LTCF	Retain		2.1 The epidemic is spreading rapidly in LTCFs
	3.2 It will be difficult to cure if older adults were infected by acute respiratory infections	Delete		
	3.3 Older adults in LTCFs are still facing the risk of acute respiratory infections	Modify	Unclear object reference	2.2 LTCFs are still facing epidemic risks
	3.4 The risk management will be difficult, if acute respiratory infections occurred in LTCF	Delete		
	3.5 Older adults are impossible to be infected because LTCFs have been ready to prevent and control acute respiratory infections	Modify	Likely to generate ambiguity	2.3 If older adults in the facility are infected with acute respiratory infections, the LTCF I work in can effectively deal with it
4. Susceptibility		Retain		4. Susceptibility
	4.1 Older adults are the most likely to be infected	Merge	Repetitive content	3.1 Older adults are more likely to be infected with acute respiratory infections than other age groups
	4.2 Older adults are the most likely to be infected than any other populations	Merge	Repetitive content	
	4.3 The preventive measurements taken by our facility are sufficient to prevent older adults from infected by acute respiratory infections	Modify	Likely to generate ambiguity	3.2 The current epidemic prevention measures of the LTCF I work for still need improvement
	4.4 The negligence may happen even if the preventive measures are strictly taken	Modify	Likely to generate ambiguity	3.3 I think that in the environment where I work, older adults still have the risk of acute respiratory infections infection
5. Coping behavior		Delete		It does not belong to the risk of acute respiratory infections infection in older adults
	5.1 I will wear a mask when approaching people outside or go out of the facility	Delete		
	5.2 I will wash my hands before and after touching older adults	Delete		
	5.3 I will reduce the use of public transport	Delete		
	5.4 I will stay away from crowded places	Delete		
	5.5 I have ever postponed or canceled unnecessary social events	Delete		
	5.6 I reduced the opportunities to go out	Delete		

In the second round of Delphi consultation, we repeated the process established in the first round. After thorough evaluation and discussion, consensus was achieved among all experts. As a result, an initial scale was developed, comprising three dimensions and nine items (Table 6).

**Table 6:** The initial scale.

Dimension	Item
1. Severity	The harm caused by acute respiratory infections in older adults is more severe than in other age groups
	Once someone in a LTCF is infected with an acute respiratory infection, the consequences can be more severe than elsewhere
	If the epidemic in LTCF is not controlled in time, its spreading will be very fast
2. Controllability	Once an older adult is infected with acute respiratory infections, it may cause other personnel to be infected in the facility
	If there are older adults in the facility infected with acute respiratory infections, the facility I work in can effectively deal with it
	The current epidemic prevention measures of the LTCF I work in can reduce the risk of older adults contracting acute respiratory infections
3. Susceptibility	Older adults are more likely to contract acute respiratory infections disease than other age groups
	LTCFs are still at risk of the acute respiratory infection epidemic
	I feel that the capacity of the care workers to prevent the acute respiratory infections in the LTCF needs to be strengthened

### 3.2 Pre-test

Five caregivers from LTCFs were invited to evaluate the 9-items scale to assess the clarity and comprehensibility of the items. All the respondents demonstrated a strong understanding of the items, indicating that the questions were clear and effectively communicated. As a result, no further adjustments were deemed necessary, confirming the scale’s readiness for broader application.

### 3.3 Participants

We collected three rounds of data collection, resulting in a total of 343 valid questionnaires from an initial pool of 428 collected questionnaires, yielding a validation rate of 80.14%. Samples 1 and 2 were collected from January to February 2022, while Sample 3 was gathered from August to September 2024. Among the respondents, 16.91% were male, with the median age of the participants ranging between 41 and 50 years (Table 7). Sample 1 consisted of 123 participants and was employed for item analysis and exploratory factor analysis (EFA). Sample 2 included 120 participants and was employed for confirmatory factor analysis (CFA), calculation of Cronbach’ *α* coefficient, and construct validity. Sample 3 served as a supplementary collection to address the insufficient sample size of Sample 2 for conducting CFA.

**Table 7:** Demographic information of participants (*n* = 343).

Item	Group	*N*	Percentage
Gender	Male	58	16.91%
	Female	285	83.09%
Age	18~	45	13.12%
	26~	25	7.29%
	31~	58	16.91%
	41~	137	39.94%
	51~	75	21.87%
	61~70	3	0.87%
Education	Primary school or below	44	12.83%
	Middle school	123	35.86%
	Senior high school or technical secondary school	61	17.78%
	Junior college	73	21.28%
	Undergraduate or above	42	12.24%
Marital status	Unmarried	55	16.03%
	Married	264	76.97%
	Divorced	24	7.00%
Working years in LTCF	< 1	70	20.41%
	1~	190	55.39%
	6~	52	15.16%
	10~	31	9.04%
Charge of the LTCF (*yuan* per month)	0 ~ 2000	39	11.37%
	2000~	128	37.32%
	4,000~	109	31.78%
	6,000	51	14.87%
	8,000~	16	4.66%

### 3.4 Item analysis

The results revealed that the absolute t-values for each item in the scale were all exceeded 3 (*p* < 0.05) (Table 8), which indicated that all items had satisfactory discriminatory power and should be retained.

**Table 8:** Item analysis (sample 1, *n* = 123).

No.	Group		*t*
	High score	Low score	
1	5 ± 0	4.46 ± 0.852	−3.769*
2	5 ± 0	4.4 ± 0.914	−3.884***
3	5 ± 0	4.54 ± 0.817	−3.311*
4	4.98 ± 0.149	3.94 ± 1.235	−4.928***
5	5 ± 0	4.46 ± 0.817	−3.932***
6	4.98 ± 0.149	3.89 ± 1.078	−5.947***
7	5 ± 0	4.09 ± 1.011	−5.351***
8	4.89 ± 0.318	3.06 ± 1.282	−8.258***
9	4.89 ± 0.318	3.8 ± 1.106	−5.645***

*P < 0.05; ***P < 0.01.

### 3.5 Exploratory factor analysis

The Kaiser-Meyer-Olkin (KMO) value of all items in the scale was 0.746, which exceeds the recommended cut-off value of 0.50. Additionally, Barlett’s test yielded a statistic of 318.241 (*p* < 0.001), indicating that the risk perception scale is qualified for EFA.

EFA was conducted using principal component analysis with the maximum variance method, resulting in the extraction of three factors (Table 9). The factor loadings for each item were all greater than 0.55, and the total variance explained by these factors was 68.366%. This suggests that the scale effectively captures the underlying constructs related to risk perception.

**Table 9:** Exploratory factor analyses for the RPSACL (sample 1, *n* = 123).

No.	Item	Factor loadings		
		Severity	Susceptibility	Controllability
1	The harm caused by acute respiratory infections in older adults is more severe than in other age groups	0.893		
2	Once someone in a LTCF is infected with an acute respiratory infection, the consequences can be more severe than elsewhere	0.879		
3	If the epidemic in LTCF is not controlled in time, its spreading will be very fast	0.786		
4	Once an older adult is infected with acute respiratory infections, it may cause other personnel to be infected in the facility		0.625	
5	If there are older adults in the facility infected with acute respiratory infections, the facility I work in can effectively deal with it		0.724	
6	The current epidemic prevention measures of the LTCF I work in can reduce the risk of older adults contracting acute respiratory infections		0.703	
7	Older adults are more likely to contract acute respiratory infections disease than other age groups		0.746	
8	LTCFs are still at risk of the acute respiratory infection epidemic			0.816
9	I feel that the capacity of the care workers to prevent the acute respiratory infections in the LTCF needs to be strengthened			
			0.811	
Eigenvalue		3.409	1.507	1.238
Explained variance		28.367	23.094	16.905
Cumulative variance		28.367	51.461	68.366

### 3.6 Confirmatory factor analysis

**Figure 2:**
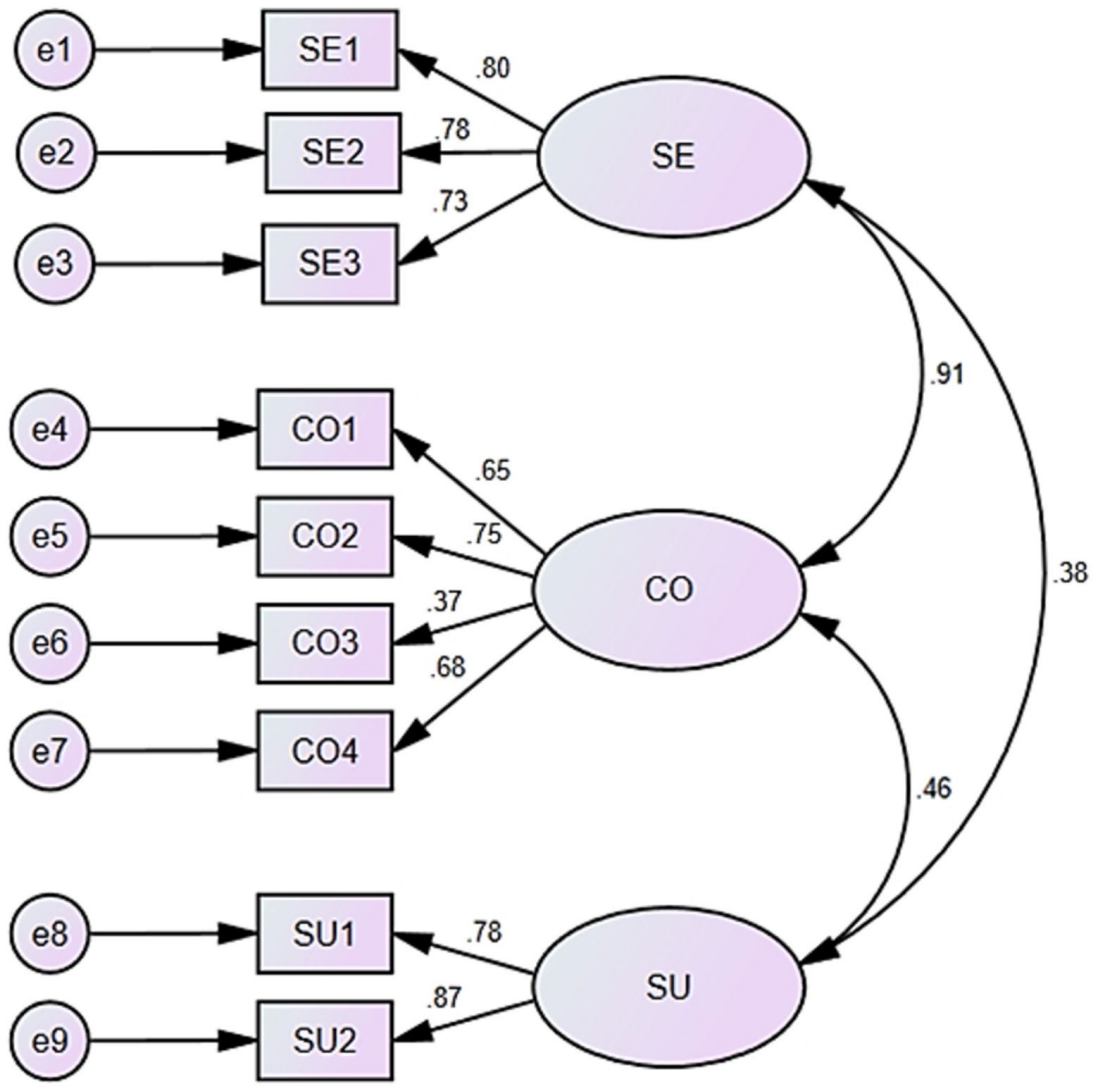
Three—factor confirmatory factor analysis model. SE, severity; CO, controllability; SU, susceptibility.

The model fit statistics of the model developed by CFA were as follows: χ^2^/DF _(120)_ = 3.800, with a NFI of 0.810, an IFI of 0.852, a CFI of 0.848, and a RMSEA of 0.153 (Figure 2). These results suggest that the model fit is not satisfactory.

### 3.7 Reliability and stability

The Cronbach’s *α* coefficient of the RPSACL was 0.795, which indicated that the whole scale demonstrates acceptable reliability. The Cronbach’s α coefficients for the three extracted factors were 0.795, 0.707, and 0.791, respectively.

The Pearson correlation coefficient between the two sets of responses was 0.564 (*p* < 0.01), indicating a moderate degree of stability within the scale. This suggests that the RPSACL is not only reliable but also stable over time, reinforcing its utility in measuring risk perception.

### 3.8 Convergent validity and discriminant validity

To assess the convergent validity of the dimensions within the scale, both the composite reliability (CR) value and the square root of average variance extracted (SAVE) value were calculated. The result indicated that the AVE for each dimension was greater than the correlation values with other factors (Table 10). This finding suggests that each dimension not only demonstrates good convergent validity—indicating that the items within each dimension are measuring the same underlying construct—but also shows strong discriminant validity, as the dimensions are sufficiently distinct from one another. Overall, these results support the robustness and validity of the scale in measuring the intended constructs.

**Table 10:** The convergent validity and discriminant validity (sample 2, *n* = 120).

	AVE^①^	CR^②^	SE^④^	CO^⑤^	SU^⑥^
SE	0.596	0.816	1	—	—
CO	0.416	0.732	0.141***	1	—
SU	0.685	0.813	0.112*	0.177***	1
SAVE^③^	—	—	0.772	0.645	0.828

*p < 0.05; ***p < 0.01.

① AVE, average variance extracted.

② CR, composite reliability.

③ SAVE, square root of the AVE.

④ SE, severity.

⑤ CO, controllability.

⑥ SU, susceptibility.

### 3.9 Further improvement of the scale structure

**Figure 3:**
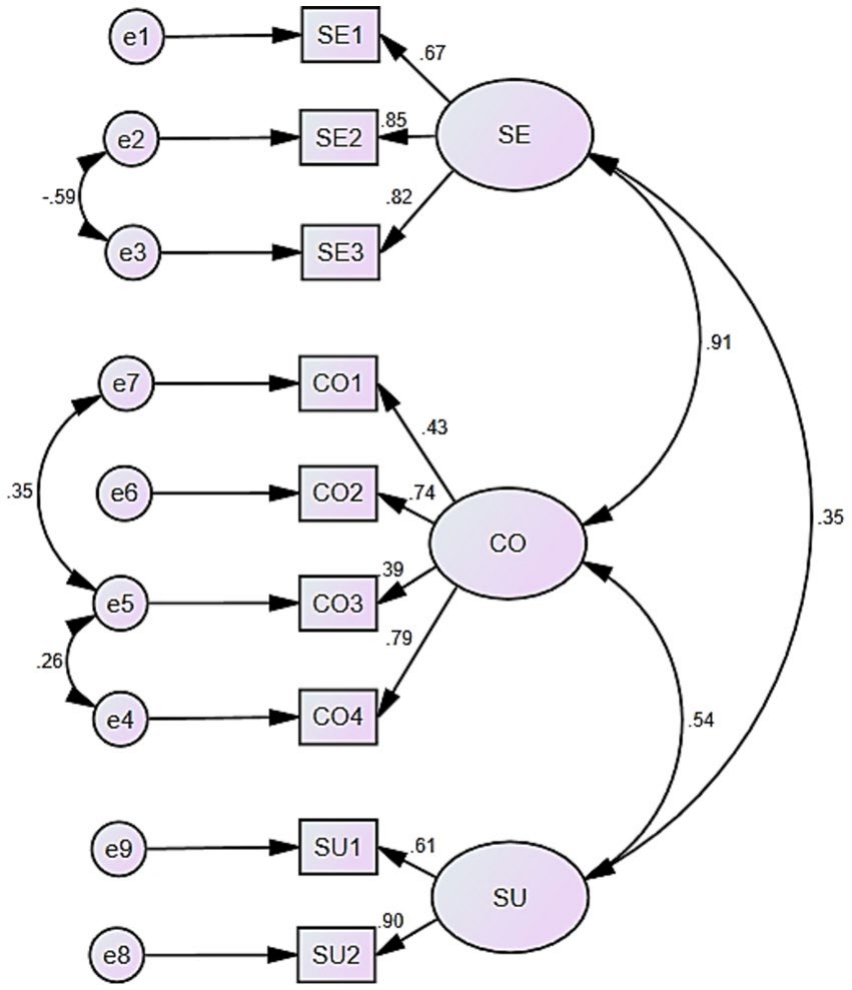
Three—factor confirmatory factor analysis model with larger sample size. SE, severity; CO, controllability; SU, susceptibility.

Given the suboptimal fit of the confirmatory factor analysis model, we systematically explored multiple approaches to resolve this limitation. According to Kyriazos’ research (44), we contributed it to insufficient sample size. To rectify this, we collected an additional 100 samples from August to September 2024. Subsequently, we merged newly collected samples with those from the previous CFA, resulting in a total of 220 samples. We then re-executed the CFA, which significantly improved model fit: χ^2^/DF _(220)_ = 2.998, with a Normed Fit Index (NFI) of 0.913, an Incremental Fit Index (IFI) of 0.940, a Comparative Fit Index (CFI) of 0.939, and a Root Mean Square Error of Approximation (RMSEA) of 0.096 (Figure 3). These results indicate the model fit is now acceptable.

## 4 Discussion and implications

RPSACL, aiming to measure risk perception of infection of care recipients, is shown to have adequate psychometric properties to apply to caregivers. Moreover, there is no existing instrument available that has the same characteristics as the RPSACL. Caregivers’ perception of risk can directly influence their coping behaviors, which in turn affect the health of older adults during ARIs outbreaks (17). Thus, understanding and measuring caregivers’ risk perception through tools like the RPSACL is vital for enhancing protective measures and safeguarding the health of older adults in these vulnerable settings.

This study represents an innovative explorations of risk management within long—term care facilities, focusing specifically on caregivers who support vulnerable populations with limited self-care capabilities. Protective behaviors exhibited by caregivers are essential in preventing the spread of ARIs within LTCFs. Previous research showed the significance of risk perception in promoting caregivers’ protective behaviors (45), highlighting its critical role in the prevention of ARI transmission. Through the integration of risk perception with the role and responsibilities of caregivers, the research enriches both the conceptual understanding and practical application of risk perception in this context.

Despite a number of related ARI risk perception scales developed, such as the Pandemic Risk Perception Scale (PRPS) (46), the public health emergency risk perception scale (PHERPS) (47), and the Questionnaire on Perception, Risk, Coping, Knowledge, COVID-19 (PRCK-COVID-19) (48), none of these instruments adequately address the specific needs of measuring the risk perception among caregivers in LTCFs. These existing scales primarily focus on the potential risks encountered by the general public in common public settings and their coping intentions, rather than the unique context in which caregivers operate. Caregivers in LTCFs face unique challenges. Given their work environments and the needs of care recipients, risk perception assessment must be tailored to them. There is a need for an assessment tool that accurately reflects caregivers’ risk perceptions and protective intentions. In LTCFs, caregivers must manage their own risks and proactively reduce infection risks for care recipients. This dual responsibility underscores the importance of acknowledging protective intentions in risk assessment. The lack of attention to these factors in existing scales hinders the ability to conduct targeted assessments that are crucial for developing effective risk management strategies tailored to the needs of caregivers and the vulnerable populations they serve.

Moreover, the study provides new insights into understanding and addressing public health risks within LTCFs. Notably, it addresses a significant gap in the existing literature by developing a specialized risk perception scale tailored specifically for caregivers, as current assessment instruments have not adequately met this need. Utilizing a rigorous psychometric methodology, the newly established scale facilitates a comprehensive evaluation of caregivers’ perceptions regarding ARI risks confronted by care recipients. By providing LTCFs with this essential tool, the research equips LTCFs with a critical tool for implementing more refined and effective risk management strategies, ultimately enhancing the safety and well-being of older adults in these care settings.

Based on previous studies about risk perception and Delph method, this study specifically addresses the particular context of caregiver in LTCFs and developed RPSACL. This scale aims to accurately assess caregivers’ perceptions of risk related to acute respiratory infections (ARIs) by integrating elements of their work responsibilities and explicitly defining the concept of risk perception within this population. To ensure the scale’s robustness and validity, a systemic development and evaluation process was undertaken, which included item analysis, exploratory factor analysis, confirmatory factor analysis, reliability test, and construct validity. This comprehensive approach utilized data collected from caregivers employed in LTCFs, ultimately contributing to a more nuanced understanding of risk perception in this critical workforce and providing a valuable tool for enhancing infection control measures in these settings.

This study employed small group analysis for item analysis, revealing a significant correlation between the high and low score group, which justified the retention of all items. Following the establishment of criteria for factor analysis, an exploratory factor analysis (EFA) was conducted carried out involving maximum variance method. The EFA results indicated that the identified factors accounted for 68.366% of the variance in the data, suggesting acceptable interpretability of the sample. Specifically, item No.7, which states that “older adults are more likely to contract acute respiratory infections disease than other age groups,” was assigned to factor 2 based on the EFA findings. After extensive discussions with the expert panel and careful consideration, we decided to align with the EFA results and reposition item No.7 within the second dimension. As a result, the revised scale, consisting of three dimensions and nine items, which were subsequently used for further evaluation. Drawing from a comprehensive literature review, leveraging specialized professional expertise, and incorporating suggestions from the expert panel, we identified three factors: severity, controllability, and susceptibility, respectively (47, 49).

After necessary modification, the final scale contains nine items and three dimensions (Table 11). The dimension of severity includes three items. This dimension assesses caregivers’ perceptions regarding the potential consequences of ARI spread in LTCFs (50). The dimension of controllability containing four items aims to evaluate individuals’ perceptions of the LTCF’s capacity to manage and control ARI outbreaks (51), which is an important influencing factor of risk perception (52). Lastly, the dimension of susceptibility includes two items. These items assess caregivers’ preliminary judgments regarding the likelihood of older adults contracting ARIs within LTCFs.

**Table 11:** The risk perception scale on acute respiratory infections for caregivers in long-term care facilities.

**Dimension**	**Item**
Severity	The harm caused by acute respiratory infections in older adults is more severe than in other age groups
	Once someone in a LTCF is infected with an acute respiratory infection, the consequences can be more severe than elsewhere
	If the epidemic in LTCF is not controlled in time, its spreading will be very fast
Controllability	Once an older adult is infected with acute respiratory infections, it may cause other personnel to be infected in the facility
	If there are older adults in the facility infected with acute respiratory infections, the facility I work in can effectively deal with it
	The current epidemic prevention measures of the LTCF I work in can reduce the risk of older adults contracting acute respiratory infections
	Older adults are more likely to contract acute respiratory infections disease than other age groups
Susceptibility	LTCFs are still at risk of the acute respiratory infection epidemic
	I feel that the capacity of the care workers to prevent the acute respiratory infections in the LTCF needs to be strengthened

This study conducted CFA to evaluate the scale. The model revealed that the overall model fit could be improved, as the RMSEA value exceeds the commonly accepted threshold of 0.10 (36). To improve the model fit, we tried to find out the main causes and contributed it to be insufficient sample size (44). Then, we procured an additional 100 responses. Integrating these with the previously gathered 120, we amassed a total of 220. All the questionnaires underwent CFA in a unified dataset. Eventually, the model fit got significantly improved with lager sample size. Additionally, The Cronbach’s *α* coefficients of three dimensions and the overall scale exceed 0.7, suggesting that each factor and the overall scale exhibit a satisfactory level of internal consistency (20). Moreover, the RPSACL showed moderate stability, and it also showed good convergent and discriminant validity between each dimension in scale evaluation.

In the context of global aging, long-term care facilities (LTCFs) are increasingly diverse, encompassing various cultural regions with distinct caregivers and care recipients. When applying measurement scales, cross-cultural adaptability becomes essential. The RPSACL scale utilized in this study is derived from data collected from nursing staff in Chinese LTCFs; however, future research should aim to extend its applicability across different cultural contexts. Cultural differences significantly impact how individuals perceive disease severity, controllability, and susceptibility. For example, in collectivist cultures, nurses may be more inclined to depend on institutional support. While in individualist cultures, there may be a greater emphasis on personal coping strategies. To assess the RPSACL scale’s cross-cultural applicability, we need to gather data from nurses of diverse cultural backgrounds, considering regional nursing practices, cultural norms, and disease perspectives. By comparing data across different cultural groups, researchers can evaluate the scale’s reliability and validity, allowing for necessary adjustments to ensure accurate measurement of acute respiratory infection (ARI) risk perception among nurses from diverse cultural settings. This approach will not only enhance the scale’s utility but also contribute to a more comprehensive understanding of ARI risk perception in the global context of long-term care.

In prospective application scenarios, managers of long-term care facilities could consider implementing the following strategies to incorporate the RPSACL scale into their risk management frameworks. First, regularly use the scale to assess nurses’ ARI risk perception. Design targeted training for those with low risk perception. For those with high risk perception but possess inadequate coping skills, specialized skill—enhancement programs should be offered to improve their ability to manage these risks effectively. Secondly, the assessment results should be comprehensively integrated into daily management processes. This includes optimizing infection prevention and control measures, rationally adjusting staff allocation, material stockpiling strategies, and work procedures in accordance with the assessment findings. Additionally, an efficient feedback mechanism should be established to continuously monitor the evaluation outcomes, thereby enabling the timely adaptation of management strategies. By implementing these measures, it is anticipated that the LTCFs will achieve a significant improvement in ARI prevention and control efficacy, thereby effectively safeguarding the health and safety of care recipients.

## 5 Limitations

Nevertheless, this study has several limitations. The RPSACL scale has primarily been employed as a preliminary measurement tool for managers to assess caregivers’ risk perceptions of acute respiratory infections (ARIs) within long-term care facilities (LTCFs), rather than serving as a comprehensive evaluation approach. Additionally, due to cultural differences across various countries and regions, the RPSACL is currently applicable to caregivers in Mainland China. Therefore, further studies are necessary to determine its validity and reliability in different cultural contexts. Future research should focus on testing the scale in a larger and more diverse population to assess its cross-cultural applicability and make any necessary adjustments to ensure it accurately reflects the risk perceptions of caregivers in other countries or regions. This will help to establish a more robust understanding of ARI risk perception globally and enhance the effectiveness of risk management strategies in LTCFs worldwide.

## 6 Conclusion

This study developed and validated a risk perception scale tailored for caregivers in LTCFs through a systematic process. The risk perception of caregivers in older adult care institutions encompasses three dimensions: severity, controllability, and susceptibility. It demonstrates that the RPSACL exhibits strong reliability and validity. Managers of LTCFs can utilize this scale to regularly assess and gain insights into the risk perception levels of their caregivers, enabling them to refine and develop strategies for epidemic prevention and formulate targeted employee training programs. Moreover, researchers can leverage this scale to investigate the risk perception status of caregivers in specific regions or during particular timeframes within LTCFs, as well as to explore its interaction with caregiver behaviors. Ultimately, these efforts will contribute to preventing the spread of ARIs in LTCFs, enhancing the quality of care provided, and promoting the overall health of older adults.

## Data Availability

The raw data supporting the conclusions of this article will be made available by the authors, without undue reservation.

## Footnotes

^1^ www.wjx.cn
