# Supplementary material for: Development and validation of a risk perception scale on acute respiratory infections for caregivers in long-term care facilities
Source: Front Public Health. 2025 May 2;13:1527905. doi: 10.3389/fpubh.2025.1527905 (PMC12087014; doi:10.3389/fpubh.2025.1527905)
Supplement: Supplementary file 1 [file Data_Sheet_1.pdf]

Supplementary 1

Method for Calculating Expert Authority Coefficient

1. Expert authority

(1) Academic standard weight (q1). (Table 1)

Table 1 Academic Standard Weight

Title	The weight to academic standards
Senior	1.0
Vice- senior	0.8
Intermediate	0.6
Junior	0.4

(2) Judgment weight (q2) = T+E+L+F. (Table 2)

Table 2 Judgment Weight

Judgment	The Impact on Expert Evaluation		
	High	Medium	Low
Theoretical analysis（T）	0.25	0.2	0.15
Experience（E）	0.25	0.2	0.15
Literature（L）	0.25	0.2	0.15
Feeling（F）	0.25	0.2	0.15

(3) Familiarity weight (q3) ranges from very familiar to not familiar at all, divided into 5 levels. Each expert assesses their level of familiarity across various dimensions. (Table 3)

Table 3 Familiarity Weight

The level of familiarity	q3
Very Familiar	1
Familiar	0.8
Commonly Familiar	0.6
Not Very Familiar	0.4
Not Familiar	0.2

(4) Expert authority coefficient = (q1+q2+q3)/3. Overall expert authority coefficient = ∑ Expert authority coefficient/Number of experts.
